# Factors associated with the critical thinking ability of professional nurses: A cross‐sectional study

**DOI:** 10.1002/nop2.875

**Published:** 2021-04-05

**Authors:** Tuan Van Nguyen, Hsueh‐Erh Liu

**Affiliations:** ^1^ Faculty of Nursing and Medical Technology Can Tho University of Medicine and Pharmacy Can Tho Vietnam; ^2^ School of Nursing College of Medicine Chang Gung University Taoyuan Taiwan; ^3^ Department of Rheumatology Chang Gung Memorial Hospital Linkou Taiwan; ^4^ Department of Nursing College of Nursing Chang Gung University of Science and Technology Taoyuan Taiwan

**Keywords:** critical thinking, nursing, nursing care, Vietnam

## Abstract

**Aim:**

To measure the level of critical thinking among Vietnamese professional nurses and to identify the related factors.

**Design:**

A cross‐sectional design was used.

**Methods:**

The total sample included 420 professional nurses. Data were collected from July to September 2019 in three public hospitals located in Southwestern Vietnam. The level of critical thinking was measured using the Vietnamese version of the Nursing Critical Thinking in Clinical Practice Questionnaire. The data were analysed using the independent Student's *t* tests, ANOVA, Pearson's correlation and regression analysis.

**Results:**

Most of the participants had a low (48.3%) or moderate (45.5%) level of critical thinking. Age, gender, ethnicity, education level, health condition, duration of working as a nurse, duration of working in the current hospital, having heard the term “critical thinking” and work position had an impact on the critical thinking ability. Work position and gender explained 11% of the total variance in critical thinking ability.

## 1 INTRODUCTION

Critical thinking is defined as the cognitive process of reasoning that involves trying to minimize errors and to maximize positive outcomes while attempting to make a decision during patient care (Zuriguel‐Pérez et al., 2015). The importance of critical thinking in nursing practice has been identified in the literature (Chang et al., 2011; Ludin, 2018; Mahmoud & Mohamed, 2017; Yurdanur, 2016; Zuriguel‐Pérez et al., 2015). The current nursing environment has become more complex and demanding, especially regarding the acuity and safety of patients and the rapid turnover rate of hospitalization. If professional nurses want to provide high‐quality care, critical thinking is required (Berkow et al., 2011; Brunt, 2005; Fero et al., 2009; Zuriguel‐Pérez et al., 2015). Nurses are often the first‐line professionals to observe and provide direct care for patients. Therefore, critical thinking is a necessary skill for them to be able to analyse clinical situations in order to make fast and correct decisions (Lee et al., 2017). More importantly, critical thinking can also improve patient outcomes by preventing habitual thinking that may lead to incorrect medication or procedures (Fesler‐Birch, 2005). The critical thinking ability of nurses can have an impact on the patient's safety, and it is a priority in educational programs for healthcare providers (Berkow et al., 2011; Buerhaus et al., 2006). We can identify those with poor critical thinking and provide in‐service education. Although critical thinking has been shown that is influenced by the experience and knowledge acquired during clinical practice (Zuriguel‐Pérez et al., 2015), other personal information needs to be considered to clarifying. Therefore, it is essential to measure the levels of critical thinking and to identify the work‐related and personal‐related factors that influence the critical thinking of nurses.

## 2 BACKGROUND

The literature has identified that there is a relationship between leadership and positive patient outcomes, such as fewer medication errors and nosocomial infections, lower patient mortality and higher patient satisfaction (Van Dyk et al., 2016; Wong, 2015). Alongside leadership, critical thinking is an important factor that supports the management. They can apply critical thinking skills in decision‐making and problem‐solving, and they can develop strategies that help staff nurses to improve their critical thinking ability (Van Dyk et al., 2016; Wong, 2015; Zuriguel‐Pérez et al., 2018). Thus, the ability to think critically is necessary for nurses because it will help them to effectively make decisions and to solve problems in practice.

Although the importance of critical thinking in nursing practice has been identified, a limited number of studies have been conducted in this population. Particularly, few hospitals have evaluated the critical thinking skills of nurses before employment or during the clinical competency evaluation (Lang et al., 2013). By reviewing 90 articles to assess the current state of the scientific knowledge regarding critical thinking in nursing, Zuriguel‐Pérez et al., (2015) found that only 16 studies used working nurses as participants. Furthermore, Zuriguel‐Pérez et al., (2018) reported that few studies have explored the critical thinking ability of nurse managers (NMs). Moreover, several studies have identified that working nurses have a low (Lang et al., 2013; Yurdanur, 2016) or moderate level of critical thinking (Chang et al., 2011; Lang et al., 2013; Zuriguel‐Pérez et al., 2018). To the researchers’ knowledge, no studies have investigated this issue in Vietnam.

In order to improve the quality and safety of patient care, various types of professional nurses have been established, such as Registered Nurses (RNs), NMs and administrative assistants (AAs). RNs provide direct care to the patients, NMs are responsible for forwarding management and delivering expert clinical care for patients, and AAs are an integral part of maintaining the quality of patient care. The AAs perform administrative tasks (e.g. filing, taking meeting minutes and distributing them and undertaking regular reports) that help NMs to spend more time assisting staff nurses and taking care of patients (Locke et al., 2011). Therefore, RNs, NMs and AAs need to cooperate to help patients to regain their health.

In Vietnam, professional nurses work in three different positions, which are NMs, general nurses (GNs) and AAs (Ministry of Health, 1997). Specifically, NMs are recognized as head nurses in Western countries, and their responsibilities are in charge of organizing and implementing comprehensive patient care and conduct a variety of administrative work (e.g. planning and assigning work to nurses, planning the acquisition of tools and consumables, checking care sheets, recording daily labour). GNs are similar to RNs in Western countries, and they provide direct and comprehensive care to patients. AAs perform administrative tasks (e.g. keeping records about the hospitalized and discharged patients, preserving medical records, managing daily medications). They also participate in patients care if necessary (Ministry of Health, 1997, 2011). Although the roles of these three types of professional nurses are different, their final goal is the same to provide holistic care for patients. With the cooperation and effort of these three types of professional nurses, patients can recover. Therefore, more surveys are needed that examine these participants’ level of critical thinking and the associated work‐related factors.

Previous studies have also found that several personal‐related factors are associated with the nurses' critical thinking ability, which are age, gender, ethnicity, education qualification, working experience and shift work (Chang et al., 2011; Feng et al., 2010; Howenstein et al., 1996; Lang et al., 2013; Ludin, 2018; Mahmoud & Mohamed, 2017; Ryan & Tatum, 2012; Wangensteen et al., 2010; Yildirim et al., 2012; Yurdanur, 2016; Zuriguel‐Pérez et al., 2018). However, the relationships between the critical thinking ability and these variables are inconsistent. For example, age and critical thinking have been found to be positively correlated (Chang et al., 2011; Ludin, 2018; Zuriguel‐Pérez et al., 2018), negatively correlated (Howenstein et al., 1996) and not related (Lang et al., 2013; Mahmoud & Mohamed, 2017; Yurdanur, 2016). Gender and critical thinking have been reported with a statistically significant relationship (Liu et al., 2019; Ludin, 2018) and no relationship (Mahmoud & Mohamed, 2017; Wangensteen et al., 2010). Level of education and critical thinking have been found in a positive association (Chang et al., 2011; Ludin, 2018) and not association (Lang et al., 2013; Mahmoud & Mohamed, 2017). Year of experiences and critical thinking have been shown to be positively correlated (Chang et al., 2011; Ludin, 2018), negatively correlated (Howenstein et al., 1996) and not related (Lang et al., 2013; Mahmoud & Mohamed, 2017). Those inconsistent findings indicated the relationships between the personal‐characteristics and the critical thinking ability of professional nurses need further exploration. Therefore, this study aimed to examine the level of critical thinking of professional nurses and to explore the work‐related and personal‐related factors. This is the first study to investigate this issue in Vietnam. The results of the current study will make a significant contribution to the literature because it will provide thorough descriptions of the critical thinking of professional nurses and its associated factors. Furthermore, the findings may be used as a baseline for nurse managers and nurse educators to propose further strategies to improve this ability in professional nurses.

## 3 METHODS

### 3.1 Research design

A cross‐sectional design was used. The Strengthening the Reporting of Observational Studies in Epidemiology guidelines were applied in this report (Von Elm et al., 2014).

### 3.2 Setting and sampling

Data collection was carried out from July to September 2019 in three representative and major public hospitals located in the Southwestern region of Vietnam. These hospitals have the same organizational structure, role of treating, operation of professional nursing and provide similar quality of health care to people around that area. The total numbers of professional in these three hospitals nurses were around 1,200. Besides, our study has two steps. The first step was to translate the English version of the Nursing Critical Thinking in Clinical Practice Questionnaire (N‐CT‐4 Practice) into the Vietnamese version. In that step, we used data as a pilot study to estimate the sample size in the second step, which was reported here. Sample size calculation was done by the formula: *n* = 1.96^2^ × p × (1‐p)/0.05^2^, where *p* = .46 came from the poor level of critical thinking among nurses in the first step and 0.05 indicated the acceptable margin of error (5.0%); 382 participants were required by this formula. An additional 10% of participants were done to adjust for potential failures such as withdrawals or missing data (Suresh & Chandrashekara, 2012). Therefore, in total, 420 participants were required for this study. Convenience sampling was conducted to recruit the sample. The inclusion criteria were the nurses' employed full‐time employment in the study hospitals. Participants who participated in step 1 or being absent during the data collection such as sick leave or delivering a baby were excluded. Participants were grouped in each hospital and received an envelope with all questionnaires. Then, researchers explained the research's purpose, benefits and risks to the potential participants and the procedure for ensuring confidentiality, and the voluntary nature of the participation. The informed consent form was signed immediately after they agreed to participate in this study. Then, the participants were required to complete the questionnaires in 20 to 30 min and to return them to the data collector.

### 3.3 Data assessment

#### 3.3.1 Sample characteristics

This instrument collected data about the personal information and occupational variables. The personal information included age, gender, marital status, ethnicity, religion, education level and self‐rated health conditions. The occupational variables were the duration of working as a nurse, the duration of working in the current hospital, the duration of working in the specific position, having heard the term “critical thinking” or not, previous exposure to critical thinking training or education or not, and type of work position.

#### 3.3.2 Vietnamese version of the Nursing Critical Thinking in Clinical Practice Questionnaire ((N‐CT‐4 Practice (V‐v))

The N‐CT‐4 Practice (V‐v) was used to measure the critical thinking ability of the professional nurses. The original instrument (N‐CT‐4 Practice) was established and classified based on the four dimensions of the 4‐circle critical thinking model of Alfaro‐LeFevre (Zuriguel‐Pérez et al., 2017). These four dimensions were personal; intellectual and cognitive; interpersonal and self‐management; and technical dimensions. The personal dimension has 39 items to assess the individual pattern of intellectual behaviours; the intellectual and cognitive dimension has 44 items to assesses the knowledge of activity comprehension connected to the nursing process and decision‐making. For the interpersonal and self‐management dimension, it has 20 items to analyse interpersonal abilities that allow for therapeutic communication with patients and health teams and to gain information that is associated with the patient in the clinical environment. The final one, the technical dimension, has 6 items to is concerned with knowledge and expertise in the procedures that are part of the discipline of nursing. This scale has 109 items that are rated using a four‐point Likert response format (1 = never or almost never, 2 = occasionally, 3 = often, and 4 = always or almost always), for example: “I recognize my own emotions.” (item 1); “I have the scientific knowledge required to carry out my professional practice.” (item 40); “I adapt information to the needs and capacities of the patient.” (item 84); “I possess skills in the use of information and communication technologies needed to produce optimal professional results.” (item 105). The total score is obtained from the sum of the 109 items. The scores range from 109–436, and they are categorized into a low level (score <329), moderate level (score between 329–395) and high level (score >395). The overall Cronbach's alpha was 0.96, and the intraclass correlation coefficient (ICC) was 0.77 (Zuriguel‐Pérez et al., 2017).

The N‐CT‐4 Practice (V‐v) was translated, and its psychometric properties were tested with 545 Vietnamese nurses. The results showed that the N‐CT‐4 Practice (V‐v) has acceptable reliability (Cronbach's alpha) and validity (content and construct validity). Particularly, the overall Cronbach's alpha was 0.98, with that of the four dimensions ranging from 0.86–0.97. The ICC was 0.81 over two weeks. The item content validity index was 1.0. Moreover, the goodness‐of‐fit indexes in a confirmatory factor analysis showed acceptable values, which were χ^2^/*df* = 2.87, root mean square error of approximation (RMSEA) = 0.059, standardized root mean square residual (SRMR) = 0.063, comparative fit index (CFI) = 0.73 and Tucker Lewis index (TLI) = 0.72 (T. V. Nguyen & Liu, 2021). Therefore, the N‐CT‐4 Practice (V‐v) can be used to measure the critical thinking ability of Vietnamese professional nurses.

### 3.4 Ethical considerations

This study conformed with the ethical principles of the Declaration of Helsinki (Helsinki Declaration, 2013), and it was granted research ethics committee approval by the ethical review board of the first author's institution.

### 3.5 Data analysis

The data were analysed using SPSS for Windows version 23.0 (IBM Corp.), and both descriptive and inferential statistics were calculated. The level of significance for all analyses was set at < 0.05. First, descriptive statistics were employed to summarize the collected data. The continuous variables were described using the mean and standard deviation (*SD*), and the frequency and percentage (%) were used for the categorical variables. Next, independent Student's *t* tests, analysis of variance (with Scheffe's post hoc comparison) and Pearson's correlation analysis were conducted to explore the association between the critical thinking ability and the personal and occupational factors. Then, a multiple regression analysis using the stepwise method was performed to identify the predictors of critical thinking ability (Pallant, 2010).

## 4 RESULTS

### 4.1 Characteristics of the participants

A total of 420 participants completed the questionnaires; the characteristics of overall participants and subjects in each group are listed in Table 1. Three groups of subjects were included, which were NMs (24.8%), GNs (49.8%) and AAs (25.4%), respectively. Regarding the personal variables, almost all participants were Vietnamese (96.7%), no religion (73.1%) and had good health condition (60%). Meanwhile, the comparison among each group showed that age (*F* = 9.89, *p *< .001), gender (χ^2^ = 6.48, *p *< .05), marital status (χ^2^ = 6.77, *p *< .05) and education level (χ^2^ = 147.38, *p *< .001) had reached the statistical significance. Further analysis showed that the age of NMs was significantly older than subjects in both the GN and AA group, AA group had a higher ratio of that in the GN group, and the AA group had a higher ratio of married one than the GN group. For educational levels, subjects in the NM group had a higher ratio of bachelor and master degree, whereas the other two groups had a high ratio of diploma and associate degree.

**TABLE 1:** Characteristics of the participants (*n* = 420)

Variables	Totals		Comparisons among work position								
			NM (*n* = 104)	GN (*n* = 209)	AA (*n* = 107)	χ^2^	(1) NM	(2) GN	(3) AA	*F*‐test	Scheffe's post hoc
	*n* (%)	Mean ± *SD*	*n* (%)				Mean ± *SD*				
Personal variables											
Age (years)		32.54 ± 7.32					35.22 ± 7.08	31.46 ± 7.0	32.05 ± 7.56	9.89^***^	(1) > (2), (3)
Gender											
Male	105 (25)		28 (26.9)	60 (28.7)	17 (15.9)	6.48^*^					
Female	315 (75)		76 (73.1)	149 (71.3)	90 (84.1)						
Marital status											
Single/divorced/widowed	169 (40.2)		34 (32.7)	97 (46.4)	38 (35.5)	6.77^*^					
Married	251 (59.8)		70 (67.3)	112 (53.6)	69 (64.5)						
Ethnicity											
Vietnamese	406 (96.7)		101 (97.1)	205 (98.1)	100 (93.5)	4.79					
Other	14 (3.3)		3 (2.9)	4 (1.9)	7 (6.5)						
Religion											
No	307 (73.1)		82 (78.8)	149 (71.3)	76 (71)	2.33					
Yes	113 (26.9)		22 (21.2)	60 (28.7)	31 (29)						
Education level											
Diploma	126 (30.0)		3 (2.9)	90 (43.1)	33 (30.8)	147.38^***^					
Associate	123 (29.3)		8 (7.7)	64 (30.6)	51 (47.7)						
Bachelor's/graduate	171 (40.7)		93 (89.4)	55 (26.3)	23 (21.5)						
Self‐rated health condition											
Very good	51 (12.1)		9 (8.7)	27 (12.9)	15 (14)	6.63					
Good	252 (60.0)		71 (68.3)	126 (60.3)	55 (51.4)						
Fair/bad/very bad	117 (27.9)		24 (23.1)	56 (26.8)	37 (34.6)						
Work‐related factors											
Duration of working as a nurse (years)		9.30 ± 7.05					12.30 ± 7.12	8.08 ± 6.42	8.75 ± 7.20	13.08^***^	(1) > (2), (3)
Duration of working in the current hospital (years)		8.81 ± 6.85					11.66 ± 7.02	7.66 ± 6.33	8.29 ± 6.93	12.98^***^	(1) > (2), (3)
Duration of working in the specific position (years)		6.10 ± 5.46					5.06 ± 4.94	7.41 ± 6.21	4.05 ± 3.27	14.79^***^	(2) > (1) > (3)
Heard the term "CT"											
No	280 (66.7)		56 (53.8)	151 (72.2)	73 (68.2)	10.74^**^					
Yes	140 (33.7)		48 (46.2)	58 (27.8)	34 (31.8)						
Previous exposure to CT training/education											
No	420 (100)		104 (100)	209 (100)	107 (100)						

Abbreviations: *AA,* Administrator assistant; *CT*, Critical thinking; *GN,* General nurse; *NM,* Nurses manager*; SD*, standard deviation.

Chi‐square and one‐way ANOVA test; significant at *p < .05; **p < .01; ***p < .001.

Regarding work‐related factors, the characters of all participants and subjects in each group are also listed in Table 1. The comparison of professional experience, such as duration of working as a nurse, duration of working in the current hospital, duration of working in this specific position and heard the terminology of "critical thinking" showed a significant statistical difference among the three groups (*p *< .001). They showed that NMs had a longer duration of working as a nurse (mean = 12.30, *SD* = 7.12) and duration of working in the current hospital (mean = 11.6, *SD* = 7.02) than the other two groups; GNs had the longest duration of working in the specific position (mean = 7.41, *SD* = 6.21). More subjects in the NM group heard the terminology of "critical thinking" than subjects in the other two groups. However, none of the subjects had been exposed to critical thinking training or education. Furthermore, there was a positive correlation among age, the duration of working as a nurse, the duration of working in the current hospital and duration of working in a specific position (*r *= .78–.975, *p* < .01).

### 4.2 Level of the critical thinking of the professional nurses

The mean of the total scores of the N‐CT‐4 Practice (V‐v) for all participants was 333.86 ± 40.22 (with the average score/item = 3.06 ± 0.37), the median score was 331 (interquartile range [IQR] = 311–359), and it ranged from 204–436, which indicates that they generally had a moderate level of critical thinking. Meanwhile, most of the participants reported a low (48.3%) or moderate (45.5%) level of critical thinking. Only 6.2% of the participants had a high level of critical thinking. Regarding the four dimensions of the N‐CT‐4 Practice (V‐v), the average sum score was 119.52 ± 14.19 (with the average score/item = 3.06 ± 0.36) in the personal dimension, 136.38 ± 17.62 (with the average score/item = 3.10 ± 0.40) in the intellectual and cognitive dimension, 68.71 ± 12.65 (with the average score/item = 3.44 ± 0.63) in the interpersonal and self‐management dimension and 18.09 ± 3.01 (with the average score/item = 3.01 ± 0.50) in the technical dimension.

### 4.3 Work‐related and personal‐related factors associated with critical thinking ability

There were statistically significant associations between the critical thinking ability and some work‐related factors, such as work position (*F* = 23.30, *p* < .001), duration of working as a nurse (*r *= 0.15, *p* < .01), duration of working in the current hospital (*r* = 0.13, *p* < .05) and having heard the term "critical thinking" (*t* = −2.48, *p* < .05; Table 2). The findings indicated that NMs had higher scores than GNs and AAs. Moreover, nurses who had worked for a longer duration as a nurse or worked longer in the current hospital had a higher critical thinking ability. Meanwhile, those who had not heard the term "critical thinking" had lower scores than participants who had heard this term.

**TABLE 2:** Association between the participants’ characteristics and the critical thinking ability (*n* = 420)

Variables	Mean ± *SD*	a/b/*F*‐value	*p*‐value	Scheffe's comparison
Personal factors				
Age		0.12^a^	.**012**	
Gender				
Male	341.70 ± 37.29	2.32^b^	.**021**	
Female	331.24 ± 40.88			
Marital status				
Single/divorced/widowed	331.24 ± 40.49	−1.09^b^	.275	
Married	335.62 ± 40.03			
Ethnicity				
Vietnamese	334.57 ± 39.57	1.97^b^	.**049**	
Other	313.07 ± 53.73			
Religion				
No	334.63 ± 39.39	0.65^b^	.516	
Yes	331.75 ± 42.51			
Education level				
(1) Diploma	327.84 ± 38.20	7.45	.**001**	3 > 1, 2
(2) Associate	327.50 ± 39.25			
(3) Bachelor's/graduate	342.86 ± 40.80			
Self‐rated health condition				
(1) Very good	343.94 ± 37.25	3.41	.**034**	1 > 3
(2) Good	334.97 ± 39.47			
(3) Fair/bad/very bad	327.06 ± 42.19			
Occupational factors				
Duration of working as a nurse		0.15^a^	.**003**	
Duration of working in the current hospital		0.13^a^	.**007**	
Duration of working in the specific position		0.07^a^	.184	
Heard the term “critical thinking”				
No	330.44 ± 39.68	−2.48^b^	.**014**	
Yes	340.69 ± 40.56			
Work position				
(1) Nurse manager	355.49 ± 38.53	23.30	**<.001**	1 > 2, 3
(2) General nurse	329.11 ± 32.79			
(3) Administrative assistant	322.11 ± 46.89			

The bolded values indicate the level of statistical significance (with *p* < .05; *p* < .01; or *p* < .001) between the independent and dependent variables.

Abbreviations: *SD*, standard deviation.

^a^ Results of Pearson's correlation coefficient (*r*).

^b^ Results of the *t* test, *F* = results of the analysis of variance.

There were statistically significant associations between the critical thinking ability and some personal‐related factors, such as age (*r* = 0.12, *p* < .05), gender (*t* = 2.32, *p* < .05), ethnicity (*t* = 1.97, *p* < .05), education level (*F* = 7.45, *p* < .01) and health condition (*F* = 3.14, *p* < .05; Table 2). The findings indicated that the older nurses reported a higher critical thinking ability, and male nurses had a higher score than female ones. Vietnamese participants had higher scores than participants with other ethnicities. Participants with a bachelor's/graduate degree level of education had higher scores than participants with a diploma and associate degree level of education. Those with very good health had a higher score than participants who rated their health as fair/bad/very bad.

All of the statistically significant variables identified in the univariate analysis were selected as independent variables to determine the predictors of critical thinking ability. For the regression analysis, the categorical variables were first coded as dummy variables. The factors of having never heard of “critical thinking,” being an NM being male, being Vietnamese, having a diploma degree and being in very good health were selected as the standard factors. The results of the stepwise multiple regression method showed that there were only two predictors, namely the variables of work position and gender. Working as an AA or GN or being female can predict the critical thinking ability, and they accounted for 11% of the total variance (*F* = 17.12, *p* < .001). This indicates that the AAs and GNs had a lower level of critical thinking than the NMs. Besides, when compared with male nurses, the female nurses exhibited a lower level of critical thinking (Table 3).

**TABLE 3:** Predictors of the critical thinking ability (*n* = 420)

Model	*B*	Beta	*p*‐value	*R* square	*F*‐value	*p*‐value
Constant	362.11		**<.001**	0.11	17.12	**<.001**
Administrative assistant	−32.38	−0.351	**<.001**			
General nurse	−26.55	−0.330	**<.001**			
Female	−9.05	−0.098	.**037**			

The bolded values indicate the level of statistical significance (with *p* < .05; *p* < .01; or *p* < .001) between the independent and dependent variables.

## 5 DISCUSSION

This study showed that the critical thinking ability of most professional nurses was at a low or moderate level. This finding is consistent with previous studies (Chang et al., 2011; Lang et al., 2013; Zuriguel‐Pérez et al., 2018). Using the same tool, Zuriguel‐Pérez et al. (2018) found that the median score of the N‐CT‐4 Practice was 363 (IQR = 340–386) for clinical nurses in Spain. Our study found a slightly lower median score (331; IQR = 311–359) but it was still in a moderate level (range of score: 329–395). Although critical thinking is a relatively new issue in Vietnamese professional nurses, it is not a brand new concept. Certain elements have been included in the nursing curriculum and clinical practice (e.g. the nursing process, problem‐based learning, evidence‐based practice). Therefore, up to 66.7% of participants had never heard the term "critical thinking," but 45.5% still reported a moderate level when measured using the N‐CT‐4 Practice (V‐v).

In Vietnam, clinical professional nurses are categorized into NMs, GNs and AAs with different job descriptions. Critical thinking ability has been identified as an important component for the high quality of care around the world, except in Vietnam. In order to identify this ability, we collected data from 3 hospitals in one region and grouped these data for analysis. Based on the comparison among NMs, GNs and AAs, it was found that NMs had a higher level of critical thinking than GNs and AAs. This can be explained by the fact that NMs have a higher age, work experience and high educational qualification than the other two groups. This result partially supports the finding that NMs report a slightly higher level of critical thinking than RNs (Zuriguel‐Pérez et al., 2018). Critical thinking is a necessary skill for effective and efficient management. Evidently, at present, NMs with a high level of critical thinking create positive practice environments that can help the staff nurses to deliver high quality and safe patient care (Zori et al., 2010). Therefore, all healthcare personnel needs to learn and apply critical thinking in order to conduct their work effectively and efficiently.

For clinical nurses, continuous in‐service education is very important to update their knowledge and skill of care. Literature found various factors associated with curriculum design and learning of critical thinking ability. Therefore, grouping subjects in the present study together in order to identify the related factors could help the development of further in‐service education of critical thinking ability effectively and efficiently. In this study, a statistically significant positive correlation was found between the critical thinking ability and age, the duration of working as a nurse and the duration of working in the current hospital. These findings are consistent with previous studies. For example, older nurses have a higher level of critical thinking than younger ones (Chang et al., 2011; Chen et al., 2019; Feng et al., 2010; Ludin, 2018; Wangensteen et al., 2010; Yurdanur, 2016; Zuriguel‐Pérez et al., 2018), and nurses with more experience report a better critical thinking ability than those with less experience (Chang et al., 2011; Chen et al., 2019; Feng et al., 2010; Ludin, 2018). Older and experienced nurses are more mature in their way of thinking (Chen et al., 2019; Ludin, 2018). Because there were statistically significant positive correlations among age, the duration of working as a nurse and the duration of working in the current hospital. This indicates that older nurses have a longer duration of working as a nurse or working in the current hospital so they have better critical thinking. However, the correlation between these factors and critical thinking in the current study is small; further explorations are suggested.

This study showed that there is a significant association between critical thinking ability and gender and ethnicity, which is also supported by the literature. Ludin (2018) found that female nurses reported a lower critical thinking ability than male nurses. Traditionally, females have generally had fewer opportunities to receive education and more difficulty asserting their rights during decision‐making than males in Vietnam (L. T. Nguyen et al., 2017). Even today, the phenomenon of gender inequality still exists in certain areas in Vietnam. This traditional burden and the limited opportunities to practice in a clinical care setting might lower the levels of the female participants’ critical thinking. Ethnicity has a similar impact, as found in the present study. For example, it has been reported that Caucasian and Hispanic/Latino participants have a significantly higher critical thinking ability than African American participants (Lang et al., 2013) and that Malaysian and Indian participants report different levels of critical thinking; nevertheless, only 0.9% of the participants were Indian (Ludin, 2018). However, in the present study, as almost all of the participants were Vietnamese (96.7%), the skewed distribution of the ethnicity might limit the generalizability of the results. In future studies, an equal distribution of ethnicity is strongly recommended.

This study also confirmed that those who had a bachelor's/graduate degree had a higher level of critical thinking than those who had a diploma or associate degree, even though the former had never heard the term "critical thinking." A vast amount of studies has found that education has a positive impact on the level of critical thinking (Chang et al., 2011; Gloudemans et al., 2013; Ludin, 2018; Yildirim et al., 2012; Zuriguel‐Pérez et al., 2018). Meanwhile, this study found that participants who had heard the term "critical thinking" displayed a higher level of critical thinking than those who had not heard this term. Education might be the major reason for this variation. In the present study, only 40.7% of participants had a bachelor's/graduate degree. In order to promote their levels of critical thinking, it is necessary to arrange for them, to encourage them, to attend advanced education or to provide further content in the in‐service education.

In this study, participants with very good health had a higher level of critical thinking than participants who self‐rated their health as fair/bad/very bad. Health status does have an impact on work productivity, job performance, quality of care and extra learning (Letvak et al., 2011). Thus, poor health limits their learning and critical thinking ability. This ability is an important predictor of real‐life outcomes (e.g. interpersonal, work, financial, health and education) (Butler et al., 2017). Therefore, the causal effects between health and critical thinking ability need further exploration.

In the current study, only the female gender and the type of work position as an AA or GN were identified as predictors, and they explained only 11% of the total variance of critical thinking ability in the regression model. The uneven distribution of gender and work position might be the reason for the low variance. Even though the male was significantly less than the female, NM was fewer than GN and AA. More factors need to be included in further studies.

The limitations of this study include that it used a convenience sample from only three public hospitals located in the Southwestern part of Vietnam. This sample does not represent all professional nurses in Vietnam. The N‐CT‐4 Practice is the instrument with good psychometric properties specific for clinical practice and translated into English (Zuriguel‐Pérez et al., 2017), Persian (FallahNezhad & Ziaeirad, 2018) and Turkish (Urhan & Seren, 2019). Different points of the Likert response format were selected by tools to measure critical thinking ability. For example, the N‐CT‐4 Practice selected a four‐point Likert response and it was rated in frequency, such as 1 = never or almost never and 4 = always or almost always. However, a seven‐point Likert scale for the Critical Thinking Disposition Assessment (CTDA) was selected and rated in levels of agreement, such as 1 for very strongly disagree and 7 for very strongly agree (Cui et al., 2021). Which response format can be more reprinting the characters of critical thinking ability? Further investigation is strongly suggested. Besides, the N‐CT‐4 Practice (V‐v) questionnaire has too many items that may lead to the boredom of the participants to answer and thus affect the accuracy of the results. Moreover, the collapsing of three distinctly separate groups of nurses into one group for most of the analyses lead to not showing differences in critical thinking and influencing factors among the three groups. These factors all limit the generalization of the present results. Based on these limitations, it is suggested that the use of nationwide systematic sampling and an international comparison are strongly suggested in further studies. Regarding the critical thinking questionnaire, it would be better to use the revised versions with fewer questions. Therefore, developmental and psychometric properties are suggested to shorten this questionnaire.

## 6 CONCLUSIONS

The results demonstrate that most of the professional nurses had a low or moderate critical thinking ability. Certain personal and occupational variables were significantly associated with the level of critical thinking. Being male or working as an NM were statistically significant predictors of critical thinking ability, and they explained only 11% of the total variance.

The findings of this study indicate that it is necessary to develop strategies to improve the critical thinking ability of professional nurses. The critical thinking ability has been confirmed to be an essential factor for high‐quality health care that focuses on the quality of patient care and patient safety. Besides, providing more opportunities to pursue advanced degrees or enhancing the provision of in‐service education in hospitals that involves classroom teaching or web‐based learning is strongly recommended for this specific group of nurses. Consequently, the quality of patient care could be improved.

## CONFLICT OF INTEREST

The authors declare that they have no competing interests.

## Data Availability

The data that support the findings of this study are available from the corresponding author upon reasonable request.
